# Effects of team-based goals and non-monetary incentives on front-line health worker performance and maternal health behaviours: a cluster randomised controlled trial in Bihar, India

**DOI:** 10.1136/bmjgh-2018-001146

**Published:** 2019-08-26

**Authors:** Suzan L Carmichael, Kala Mehta, Hina Raheel, Sridhar Srikantiah, Indrajit Chaudhuri, Shamik Trehan, Sunil Mohanty, Evan Borkum, Tanmay Mahapatra, Yingjie Weng, Rajani Kaimal, Anita Sivasankaran, Swetha Sridharan, Dana Rotz, Usha Kiran Tarigopula, Debarshi Bhattacharya, Yamini Atmavilas, Wolfgang Munar, Anu Rangarajan, Gary L Darmstadt, Ananya Study Group, Y Atmavilas

**Affiliations:** 1 Department of Pediatrics and Center for Population Health Sciences, Stanford University School of Medicine, Stanford, California, USA; 2 CARE India, Patna, India; 3 Project Concern International, Delhi, India; 4 Mathematica Policy Research, Princeton, New Jersey, USA; 5 Quantitative Sciences Unit, Department of Medicine, Stanford University, Stanford, California, USA; 6 The Bill and Melinda Gates Foundation, Delhi, India; 7 Department of Global Health at the George Washington University Milken Institute School of Public Health, Washington, DC, USA

**Keywords:** community health worker, teamwork, coordination, primary healthcare, performance-based incentives

## Abstract

**Introduction:**

We evaluated the impact of a ‘Team-Based Goals and Incentives’ (TBGI) intervention in Bihar, India, designed to improve front-line (community health) worker (FLW) performance and health-promoting behaviours related to reproductive, maternal, newborn and child health and nutrition.

**Methods:**

This study used a cluster randomised controlled trial design and difference-in-difference analyses of improvements in maternal health-related behaviours related to the intervention’s team-based goals (primary), and interactions of FLWs with each other and with maternal beneficiaries (secondary). Evaluation participants included approximately 1300 FLWs and 3600 mothers at baseline (May to June 2012) and after 2.5 years of implementation (November to December 2014) who had delivered an infant in the previous year.

**Results:**

The TBGI intervention resulted in significant increases in the frequency of antenatal home visits (15 absolute percentage points (PP), p=0.03) and receipt of iron-folic acid (IFA) tablets (7 PP, p=0.02), but non-significant changes in other health behaviours related to the trial’s goals. Improvements were seen in selected attitudes related to coordination and teamwork among FLWs, and in the provision of advice to beneficiaries (ranging from 8 to 14 PP) related to IFA, cord care, breast feeding, complementary feeding and family planning.

**Conclusion:**

Results suggest that combining an integrated set of team-based coverage goals and targets, small non-cash incentives for teams who meet targets and team building to motivate FLWs resulted in improvements in FLW coordination and teamwork, and in the quality and quantity of FLW–beneficiary interactions. These improvements represent programmatically meaningful steps towards improving health behaviours and outcomes.

**Trial registration number:**

NCT03406221

**Key questions**

**What is already known?**

- Front-line community health workers (FLW) have the potential to positively impact health outcomes but face myriad challenges.
- Many questions remain regarding how best to support and motivate them and improve their coordination and performance.

**What are the new findings?**

- This cluster randomised trial combined an integrated set of team-based coverage goals and targets, small non-monetary incentives for teams who met targets and team building to motivate FLWs.
- The intervention resulted in more antenatal home visits and more mothers receiving iron-folic acid (IFA) tablets; improved attitudes related to coordination and teamwork; and increased provision of advice to mothers related to IFA, clean cord care, breast feeding, complementary feeding and family planning.

**What do the new findings imply?**

- Combining an integrated set of team-based incentives, non-monetary incentives and promotion of team-based performance should be further explored as a mechanism for improving healthcare worker effectiveness and, ultimately, health outcomes, and could augment the effectiveness of performance-based financing.

## Introduction

Front-line health workers (FLW), also referred to as community health workers, are critical contributors to primary healthcare, especially for reproductive, maternal, newborn and child health and nutrition (RMNCHN), and especially in hard-to-reach, low-resource settings.^1 2^ They typically live in the communities they serve, provide basic preventive and health-promoting services and advice, and serve as an intermediary between communities and the healthcare system. In recent years, there has been increasing focus on their contributions and reliance on them to provide health-related services^3^ and information at the ‘last mile’. FLWs have the potential to positively impact health outcomes but face myriad challenges, and many questions remain regarding how best to support and motivate them and improve their coordination and performance.^4^

Goal setting, incentives and team-based structures are known to improve job-related motivation and performance.^4–6^ Knowledge from low-income country settings is limited, but a number of studies around the world have reported that factors such as clarity of expectations, various types of monetary and non-monetary incentives, training and well-defined roles have all been associated with higher motivation and better performance among FLWs.^3^ An intervention in the Philippines reported that performance-based financial incentives for doctors resulted in improvements in children’s health.^7^ Findings from other interventions regarding financial incentives and outcomes related to maternal and child health have been somewhat mixed, and largely focus on facility-based care rather than FLWs.^8^ Few studies have directly evaluated the role of teams, but a study conducted in Mozambique reported that implementation of a team structure resulted in increased cohesion among different types of FLWs, more consistent health-related messaging and less conflict about roles and responsibilities.^9^ An intervention in Rwanda reported that financial incentives paid to health centres, which may have relevance to goal setting and incentivising teamwork, resulted in improved health outcomes related to maternal and child health.^10^

In India, the principal FLWs for provision of RMNCHN services are the Anganwadi worker (AWW), accredited social health activist (ASHA) and the auxiliary nurse midwife (ANM). Despite challenges in their coordination and teamwork, no interventions have been reported to improve their performance as a team. This paper evaluates the impact of a ‘Team-Based Goals and Incentives’ (TBGI) intervention in Bihar, one of India’s poorest and most populous states.^11^ The intervention was designed to leverage the power of incentives and lessons from motivational theory on teamwork and goal setting to improve FLW performance and, in turn, health-promoting behaviours related to RMNCHN.^4–6^ Unique features were that the intervention awarded non-monetary rather than monetary incentives based on team rather than individual performance, incentivised the achievement of a range of outcomes rather than a single outcome and integrated incentives with goal setting and team building to motivate FLWs to work as a team and to increase their interactions with beneficiaries. The intention was that the resulting improvements in teamwork and motivation would lead to improvements in outreach to the study population and more effective communication of health messages, which would in turn positively impact health behaviours and outcomes among the beneficiaries.

We evaluated the impact of this intervention on improvements in maternal beneficiaries’ behaviours that were related to the intervention’s team-based goals (primary outcomes), other RMNCHN health-related behaviours and FLW performance as measured by interactions of FLWs with each other and with maternal beneficiaries (secondary outcomes).

## Methods

### Study design: TBGI intervention

This cluster randomised controlled trial was conducted from May 2012 to November 2014 in the Begusarai district of Bihar. The intervention was introduced as a supplement to reinforce the overall objectives and health messages of the *Ananya* programme that was concurrently implemented throughout Begusarai and seven other districts in Bihar. *Ananya*’s long-term goal was to improve RMNCHN outcomes state-wide in Bihar by improving behaviours related to family planning, antenatal care and delivery preparation, postnatal maternal and newborn care, complementary feeding and child immunisation. *Ananya* included an integrated set of interventions at the household, community and health facility levels which were designed to improve health behaviours and outcomes by increasing the supply and demand for primary healthcare focused on RMNCHN. The goals included in the TBGI intervention were already integral to existing government-supported health programmes and were supported by core *Ananya* interventions at the health subcentre level across *Ananya* project districts. The estimated impacts of the TBGI intervention thus reflect the added value of the TBGI intervention in addition to core *Ananya* programme interventions. The TBGI intervention was designed by CARE India, in partnership with the Georgia Institute of Technology, and implemented by staff from the Ministry of Health and Family Welfare (MoHFW) and the Integrated Child Development Services (ICDS) within the Ministry of Women and Child Development (MoWCD) in the selected sites with facilitation from CARE field teams.

Box 1 describes the three main components of the TBGI intervention: additional detail on design of the intervention was published previously by Meyer *et al*.^4^ The intervention was aimed to foster a sense of team collective responsibility and solidarity by emphasising the value of teamwork and by having FLWs jointly author and recite a pledge at regular health subcentre meetings—an important convergence and coordination platform set up by the Government of Bihar as part of the *Ananya* programme—to serve all beneficiaries in their community. Seven goals and coverage targets (quarterly and annual) were established related to RMNCHN outcomes, and small non-cash incentives were awarded to FLW teams each quarter if their subcentre team met >70% of the collective goals for that quarter, which was determined using data from ongoing data collection. These non-cash incentives were household items such as utensils, cookware and storage containers, selected by FLWs from a catalogue of choices. Non-cash incentives also included a certificate of recognition from the District Magistrate (the highest administrative officer of the district administration) for teams that met their targets in all four quarters of the year.

Box 1 **Description of components of the Team-Based Goals and Incentives (TBGI) intervention trial in Begusarai, Bihar, 2012–2014**

**Team**

- Front-line health workers (FLW) were provided training on the importance of teamwork at monthly subcentre meetings.
- A service pledge was introduced, which reiterated the FLWs’ mission to improve the health of beneficiaries and was recited jointly by them at subcentre meetings, as follows: ‘We are vital members of our Health Sub-Center (HSC) Team. We pledge to uphold our HSC Code, thereby improving the health and well-being of our entire community, irrespective of caste, religion, or geographic distance. Achieving this mission will require hard work, cooperation, and a shared willingness to help our Sub-Center sisters at all costs. By achieving this mission, we provide a valuable service to the Nation of India, the State of Bihar, our communities, our team our families, and ourselves. We also pledge we will work with dedication and will not breach the faith of community. We will make every effort to provide services to all the beneficiaries of the community and achieve at least the given target.^4^ ’

Goals (coverage targets for year 1 and year 2 of the intervention)*

- Pregnant women arranged for transportation for their delivery (70%, 90%).
- Pregnant women received at least 90 iron and folic acid (IFA) tablets (70%, 80%).
- Children were breast fed within an hour of birth (70%, 90%).
- Deliveries included appropriate umbilical cord care (70%, 90%).†
- Children aged 6–11 months were fed age-appropriate and nutritious food (70%, 70%).‡
- Women (or their partners) used any modern method of family planning within 6 months of delivery (30%, 40%).
- Children received a DPT3 injection by age 6 months (80%, 90%).

**Incentives**

- All FLWs (ie, ASHA, AWW, ANM) in a subcentre received non-cash incentives if their subcentre met five of seven goals per quarter.
- The incentives consisted of stoves, casserole dishes, storage containers or similar household items.
- An additional prize (a pressure cooker to speed food preparation in the first year and a set of five copper-coated cooking and serving bowls in the second year) was given at the end of the year to FLWs in those subcentres that successfully met their targets in all four quarters.
- Teams that met their targets in all quarters received a certificate of recognition at an end-of-year function.

The total cost per FLW for non-cash incentives, if she met all quarterly targets and the annual target, was between $20 and $30.*For each goal, a coverage target was set for the percentage of eligible beneficiaries in the subcentre catchment area who should have adopted the relevant behaviour.†The intervention emphasised not applying any substance to the umbilical cord after cutting.‡The intervention emphasised cereal-based feeding of children 6 months and older from a separate bowl with sufficient (age-dependent) frequency and quantity.ANM, auxiliary nurse midwife; ASHA, accredited social health activist; AWW, Anganwadi worker.

**Figure 1:**
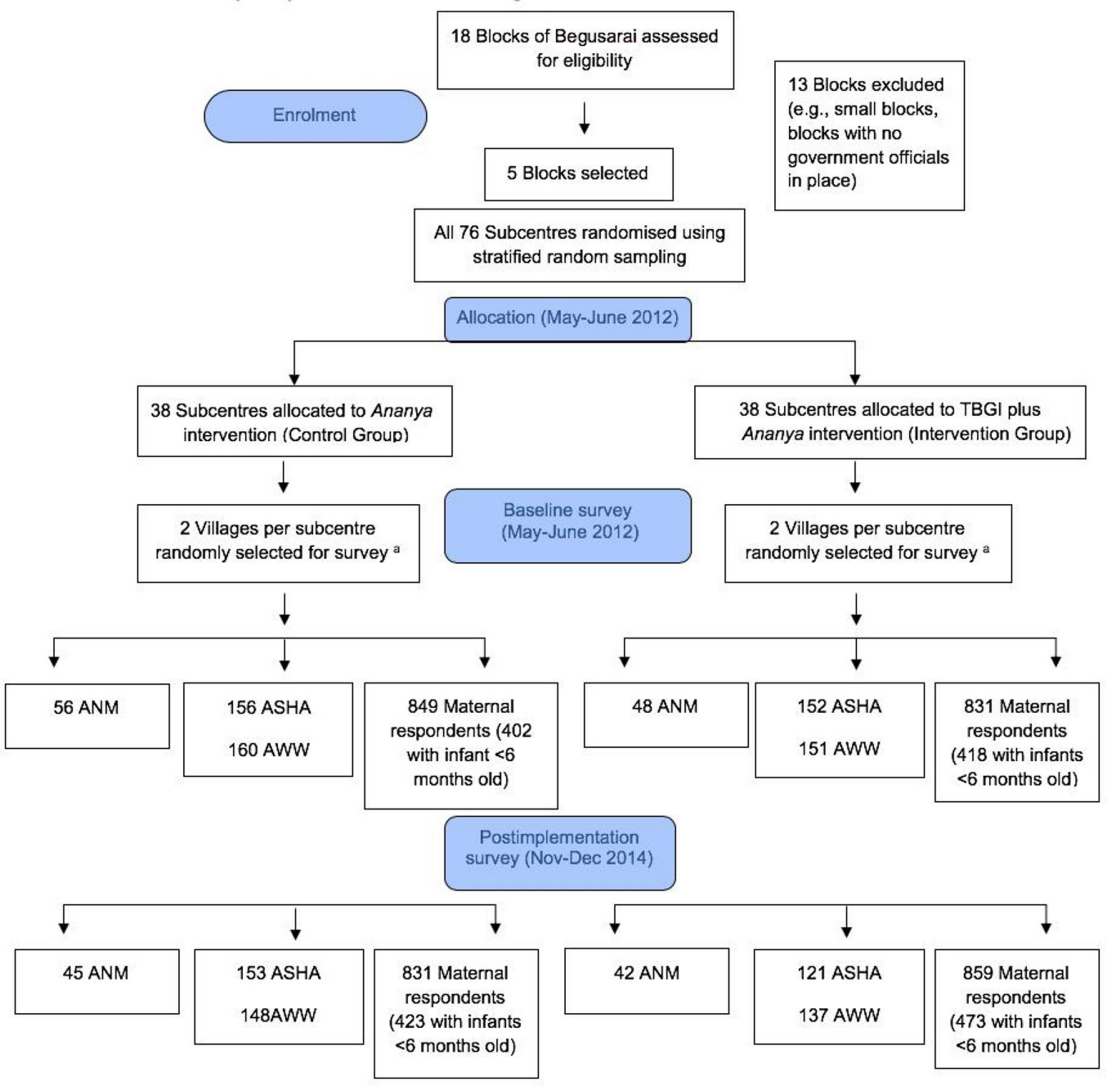
Description of the design and sample selection for the Team-Based Goals and Incentives (TBGI) intervention trail in Begusarai, Bihar, 2012–2014. (A) If a large village (population ≥150, as identified by CARE) was selected, the village was organised into approximately equal segments (75–150 households per segment) and one segment was randomly selected for surveying. ANM, auxiliary nurse midwives; ASHA, accredited social health activists; AWW, Anganwadi workers.

Figure 1 illustrates the design of the trial and its evaluation. The trial was implemented in five of 18 blocks (subdistricts) of Begusarai, which were purposefully selected to represent a range of population sizes and geographies; blocks that CARE’s field team had qualitatively determined to be atypical (such as those in which government health officials were not in place) were excluded. The five blocks which were included were all rural, and their combined population was approximately 530 000.^12^ The unit of random assignment was the health subcentre catchment area, the lowest tier of health facilities of the MoHFW. A subcentre typically serves five to six villages with a combined population of 10 000–12 000. The 76 health subcentres in these blocks were randomly assigned into equal-sized intervention and control groups (38 subcentres each) using a stratified random assignment procedure based on the number of village-level Anganwadi Centers (managed by the ICDS) linked to each subcentre, which is a proxy for the size of the population served. Specifically, subcentres in each block were divided into a stratum of ‘small’ subcentres and a stratum of ‘large’ subcentres. For each stratum, randomisation was conducted separately by assigning half the subcentres in the stratum to treatment and half to control (or about half in the case of strata with an odd number of subcentres). Sample size calculation, conducted for the purpose of designing the intervention, suggested that at least 80 subcentres were required for the study and the five selected blocks would provide the sufficient number of subcentres. Sample size estimates were performed assuming a sample of 80 subcentres and 20 women per subcentre, and an effect size of 6–10 absolute percentage point difference in the behaviours targeted by the intervention.

The primary participants in this intervention were three types of FLWs: (1) the ASHA, who supports antenatal care, delivery, newborn care and child immunisations, is paid monetary incentives by the MoHFW; (2) the AWW, who focuses on supplemental nutrition for pregnant women and young children and on preschool education, is part of the MoWCD, and is paid a salary; and (3) the ANM, who focuses mainly on antenatal care provision, delivery care, child immunisations and basic community health needs, is part of the MoHFW, and is paid a salary. While the ANM is not formally the immediate supervisor of the ASHA, the ASHA assists in mobilising clients for services provided by the ANM. Each population cluster of around 1000 persons is typically served by one ASHA and one AWW, who live in the community. The ANM provides services in and oversees activities of a health subcentre. All FLWs attending a particular subcentre constituted a single team, for the purposes of the TBGI intervention.

### Study design: intervention evaluation

We estimated impacts of the intervention using survey data collected from FLWs and a representative sample of women who had given birth in the catchment areas of the intervention and control subcentres in the year prior to each survey. The TBGI evaluation was designed and implemented by Mathematica Policy Research, which worked with Sambodhi to oversee the survey data collection. The Georgia Institute of Technology provided additional input to intervention design.^4^ The assessors who conducted the evaluation survey were unaware of which areas were part of the intervention.

Two independent cross-sectional surveys were conducted (ie, different sets of mothers were interviewed): at baseline (May to June 2012, before the intervention began) and postimplementation (November to December 2014, approximately 2.5 years after implementation began). At baseline and postimplementation, eligible mothers were interviewed using a structured questionnaire regarding home visits by the FLWs, and their health behaviours and practices related to the project’s seven goals, which were considered the primary endpoints of the intervention.

For the postimplementation survey only, ASHAs and AWWs reported on their opinions and behaviours related to teamwork, and maternal respondents reported on advice they received from FLWs related to the goals of the TBGI intervention.

Two villages per health subcentre were randomly selected for survey. In large villages (>150 households), one segment of the village was then randomly selected, with a segment which comprised about 25 eligible household respondents. All women who lived in these villages (or segments) and had given birth in the previous 12 months, and all FLWs who served these villages, were eligible for interview. Because a different cohort of women gave birth in the 12 months before each survey, and the underlying sample size was large, the baseline and postimplementation samples were likely to include different women, but they were located in the same villages, with two exceptions: two villages, which were served by two intervention and two control subcentres, could not be surveyed at follow-up due to flooding. These two villages were excluded from our final analytical cohort.

### Analysis

We compared the demographic characteristics of the ANMs, ASHAs, AWWs and maternal respondents at baseline and postimplementation according to intervention allocation, using appropriate bivariate tests (χ^2^ tests for categorical variables and t-tests for continuous variables unless otherwise noted in the tables). We then conducted a series of regression models to examine information from FLWs and mothers on indicators related to the intervention. Survey logistic regression models were conducted for binary outcomes and survey Poisson regressions for count variables.

For comparisons involving information from FLWs (which was only collected after implementation), regression models were conducted that accounted for village as the primary sampling unit and subcentre as the primary stratum within the sampling unit. Thus, SEs were adjusted for clustering at the village level. Separate regression analyses were conducted for each outcome, for each cadre (AWW, ASHA); the independent variable for these models was intervention allocation (TBGI+*Ananya* vs *Ananya* alone).

For comparisons involving information from maternal respondents, regression analyses were performed that accounted for village as the primary sampling unit and subcentre as the primary stratum within the sampling unit, and with proportional sampling weights at the maternal respondent/household level. First, we compared responses regarding types of advice mothers received from FLWs (which were only collected after implementation). Second, we compared responses regarding home visits and goal-related behaviours (which were collected at baseline and after implementation). All of these outcomes were binary. Separate regression analyses were conducted for each outcome, for each available time point; the independent variable for these models was intervention allocation (TBGI+*Ananya* vs *Ananya* alone), and the models were also adjusted for maternal age (as a continuous variable) and caste (non-Hindu; Hindu and scheduled caste/scheduled tribe (SC/ST); Hindu but not SC/ST). We then conducted difference-in-difference (DID) analyses to estimate the effect of the TBGI intervention on home visits and goal-related behaviours. These models each contained the following variables: intervention allocation (TBGI+*Ananya* vs *Ananya* alone), time (baseline or postimplementation), an interaction of allocation and time (the coefficient for the interaction term represents the DID estimator) and maternal age and caste. The DID was considered significant if p<0.10 because it represents an interaction term.^13^ P<0.05 was considered significant for non-interaction comparisons. To calculate the per cent difference between postimplementation and baseline in terms of difference of rates in the treatment versus control (also interpreted as the ‘treatment effect’), we calculated the predicted probability of each of the four groups represented by the interaction term, as appropriate for Poisson or logistic regression models.^14^ Since we had subcentre/stratum with one primary sampling unit in our data, we applied the ‘options(survey.lonely.psu=‘adjust’)’ available in the ‘survey’ package in order to obtain stable estimates in all our regressions. All analyses were performed in R V.3.4.3 via ‘survey’ package available in R CRAN (“Comprehensive **R** Archive Network”),^15^ and were reproduced independently using Stata V.14 (StataCorp. 2015. Stata Statistical Software: Release 14. College Station, TX: StataCorp). Analyses of each outcome excluded participants with missing data on that particular outcome or relevant covariates. Further details regarding the statistical methods are found in online supplementary appendix.

10.1136/bmjgh-2018-001146.supp1 Supplementary data

### Study participant involvement

FLWs provided input regarding the intervention design via preimplementation group discussions. The implementation team from CARE was in close communication with the FLWs during the intervention, ensuring their adherence to the intervention. The burden of the intervention was assessed by FLWs via the postimplementation survey. Results are being disseminated to the participating communities through the Bihar Technical Support Unit which provides guidance to implementation.

### Patient and public involvement

The development of the research question and outcome measures were informed by health and health services priorities identified by the Government of Bihar, the Bill and Melinda Gates Foundation, CARE and other collaborative organisations based on years of experience working at community level and in health facilities in the study region. In addition, intervention design was informed by formative research conducted with potential beneficiaries in the study area. FLWs provided input into the design of the study and were included in assessments; however, eligible women were not directly involved in recruitment or implementation of the study, and the burden of the intervention for beneficiaries themselves was not assessed. Results will be disseminated to policymakers, programme managers, implementers and researchers, including FLWs and governmental agencies, involved in the health sector via reports, publications and presentations. We thank the communities in the study area for their participation.

### Ethics

The trial was registered at ClinicalTrials.gov (NCT03561012). Approval for the overall *Ananya* evaluation, which included this study, was obtained from the Institutional Review Board of the Public Health Foundation of India, New Delhi. Verbal informed consent was obtained from all respondents. Data analyses were deemed exempt from oversight by the Stanford University Institutional Review Board.

### Data sharing

A technical appendix, statistical code and the data set are available upon request to the corresponding author.

## Results

Most ANMs did not live in the village where the health subcentre she managed was located, a majority were in their 40s, almost all were Hindu and most belonged to general caste and had some college-level education (table 1).^16^ In contrast, almost all ASHAs and AWWs lived in the villages they served and their median age was in the mid-30s to late 30s. Like ANMs, almost all were Hindu, but the most common caste was ‘other backward caste.’ Within each group of FLWs, these characteristics did not differ significantly between respondents from control and intervention villages, at baseline or postimplementation.

**Table 1:** Demographic characteristics of auxiliary nurse midwives (ANM), accredited social health activists (ASHA), Anganwadi workers (AWW) and maternal household respondents as part of the Team-Based Goals and Incentives intervention trial in Begusarai, Bihar, 2012–2014*

	Baseline (May to June 2012)			Postimplementation (November to December 2014)		
	Control	Intervention	P value	Control	Intervention	P value
**ANM**	n=56	n=48		n=45	n=42	
Lives in village she serves (%)	21.4	29.2	0.50	22.2	28.6	0.66
Distance from subcentre village (median and IQR, in km)†	6 (8)	5 (8)	0.48	5 (6)	8 (19)	0.12
Age (median and IQR, in years)‡	42 (7.8)	42 (15.3)	0.87	46 (16)	48 (12)	0.51
Hindu (%)	100	97.9	0.46	100	100	–
Caste (among Hindus only) (%)†						
Scheduled caste/scheduled tribe (SC/ST) (lowest caste)	8.9	4.2	0.43	8.9	9.5	0.95
Other backward caste (socially and educationally disadvantaged)	21.4	31.9		35.6	38.1	
General caste	69.6	63.8		55.6	52.4	
Attended college, took college-level courses or received diploma (%)	80.4	87.5	0.33	88.9	88.1	0.91
**ASHA**	n=156	n=152		n=153	n=121	
Lives in village she serves (%)	96.2	98.7	0.28	97.4	97.5	1.00
Age (mean and SD, in years)	35.1 (5.5)	34.4 (5.2)	0.21	35.9 (5.8)	37.3 (7.1)	0.08
Hindu (%)	95.5	97.4	0.54	97.4	97.5	1.00
Caste (Hindus only) (%)						
Scheduled caste/scheduled tribe (SC/ST) (lowest caste)	18.8	20.3	0.89	14.1	12.7	0.68
Other backward caste (socially and educationally disadvantaged)	42.3	43.2		49.7	45.8	
General caste	38.9	36.5		36.2	41.5	
**AWW**	n=160	n=151		n=148	n=137	
Lives in the village that she serves (%)	96.9	94.1	0.28	97.4	97.5	1.00
Current age (mean and SD, in years)	37.4 (7.5)	36.6 (7.0)	0.21	38.6 (7.8)	38.0 (7.1)	0.53
Hindu (%)	94.4	92.7	0.54	94.6	92.7	0.67
Caste (Hindus only) (%)‡						
Scheduled caste/scheduled tribe (SC/ST) (lowest caste)	13.9	12.9	0.97	11.4	11.0	0.63
Other backward caste (socially and educationally disadvantaged)	53.0	53.6		57.9	52.8	
General caste	33.1	33.6		30.7	36.2	
**Maternal respondents**	n=849	n=831		n=831	n=859	
Hindu (%)	88.8	89.2	0.81 0.88	89.1	90.8	0.23 0.26
Scheduled caste/scheduled tribe (SC/ST) (among Hindus only)	34.0	42.8	<0.001	30.5	39.7	<0.001
Household size (median, IQR)‡	5 (2)	5 (2)	0.44	5 (2)	5 (2)	0.73
Age (%)						
15–19 years	2.0	2.6	0.14	15.5	11.8	0.04
20–24 years	46.1	40.6		46.1	43.4	
25–29 years	38.0	39.7		27.0	30.4	
30–34 years	10.0	12.0		9.3	11.5	
35–49 years	3.9	5.1		2.2	2.9	
Age (mean and SD, in years)†	25.1 (4.1)	25.5 (4.5)	0.05	23.6 (4.3)	24.2 (4.5)	0.006
Birth parity (%)						
1 child	37.4	38.3	0.65	33.9	34.3	0.29
2 children	25.1	24.1		29.2	25.4	
3 children	19.4	17.8		19.0	20.3	
4 or more children	18.0	19.9		17.8	20.0	
Literate (%)	38.5	38.9	0.88	50.2	50.6	0.85
Below Poverty Line card (%)†	59.3	62.5	0.19	63.9	62.8	0.64
Socioeconomic status quartile§ (%)						
1	14.8	14.9	0.59	23.0	20.5	0.24
2	23.4	25.8		20.0	23.4	
3	29.8	29.8		29.2	30.2	
4	32.1	29.5		27.8	25.9	

*Results at baseline and postimplementation represent two cross-sectional samples. Results are presented without adjusting for survey design. P values reflect results from χ^2^ (categorical variables) or t-tests (continuous variables), unless otherwise noted; Fisher’s exact tests were calculated if any cell size for a comparison was <5.

†Missing data were present for some variables and are noted in this sequence: baseline control, baseline treatment, postimplementation control and postimplementation treatment group. Data were missing for ANM distance from subcentre village (12, 15, 10, 13 missing); maternal age (1, 0, 0, 0); maternal Below Poverty Line card (1, 0, 3, 2); and maternal socioeconomic status (2, 0, 1, 1).

‡Mann-Whitney U test was conducted due to non-normality.

§Socioeconomic status quartile: Lowest quartile is poorest. Quartiles were determined using coefficients and cut-offs from a principal component analysis that used the *Ananya* state-wide 2012 baseline data, following the methodology of the National Family Health Survey’s wealth index.^11^ Quartiles are therefore relative to the 2012 state-wide socioeconomic status distribution for women who gave birth in the previous 12 months.

Among maternal respondents, almost all were Hindu, about a third belonged to ‘scheduled caste or tribe’, the mean age was mid-20s, about half were literate and most had a Below Poverty Line card (table 1). As opposed to controls, women in intervention villages were significantly older and more likely to belong to a SC or ST.

Table 2 presents information about teamwork, as reported by AWWs and ASHAs after implementation. Most of these FLWs (>70%) attended subcentre meetings at least monthly on average; this frequency was significantly higher for ASHAs from intervention than control villages (87% vs 75%, p=0.02) but did not differ for AWWs (72% vs 70%). Most FLWs (>70%) considered the opposite-cadre FLW in their village to be part of their team, but there were no statistically significant differences between control and intervention area FLWs. Most FLWs also considered the subcentre ANM to be part of their team; this percentage was significantly higher for intervention than control ASHAs and AWWs (p<0.05). About 30% of FLWs considered AWWs or ASHAs who belonged to the same health subcentre but were from different villages to be part of their team; this was significantly higher for intervention than control AWWs (24% vs 13%, respectively, considered other AWWs to be part of their team) but not for ASHAs. About half or more of FLWs reported that their team was available for help when needed and for planning; percentages were higher for intervention than control FLWs but differences were not statistically significant. Expectations for meeting regularly for planning purposes with the subcentre team were higher for intervention than controls FLWs; the difference was significant for AWWs (p=0.01). AWWs and ASHAs met with ANMs outside of subcentre meetings approximately monthly; they conducted on average about 1.5 joint home visits per week; they met together about twice per week to discuss work, and they asked an opposite-cadre FLW to conduct a home visit for them about once per week, with mostly similar results for intervention and control AWWs and ASHAs.

**Table 2:** Teamwork and coordination characteristics reported by accredited social health activists (ASHA) and Anganwadi workers (AWW) after implementation in the Team-Based Goals and Incentives intervention trial in Begusarai, Bihar, 2012–2014*

Modelled indicators	AWW n=285			ASHA n=274		
	Control (n=148)	Intervention (n=137)	P value	Control (n=153)	Intervention (n=121)	P value
Attended three or more subcentre meetings in the past 3 months (%)	70.1	72.4	0.72	74.9	86.8	0.02
**Working in a team**						
Consider … part of their team						
Other FLW of the village (other cadre) (%)	87.6	90.7	0.49	80.7	70.7	0.15
Subcentre auxiliary nurse midwife (ANM) (%)	80.8	93.3	0.01	57.4	76.4	<0.01
Same-cadre front-line workers (FLW) at subcentre (%)	13.1	24.3	0.05	27.4	27.3	0.99
Other-cadre FLWs at the subcentre (%)	12.2	19.1	0.19	10.1	16.7	0.13
Can always get help from team when needed (%)	41.5	51.3	0.15	47.4	60.0	0.07
Always expected to plan with team (%)	51.1	67.9	0.01	58.4	71.4	0.08
Always expected to meet regularly with team (%)	51.8	63.7	0.08	64.4	60.5	0.52
**Working with ANM**						
Times met with ANM outside subcentre meetings in the past 3 months (mean)†	3.7	4.0	0.34	3.6	4.1	0.13
Any joint visits with ANM in the past month (%)	50.4	48.6	0.79	68.9	62.2	0.41
**Working with opposite** **-** **cadre FLW**						
Average joint home visits in the past week (mean)	1.5	1.7	0.38	1.5	1.3	0.48
Average times met with opposite-cadre FLW in the past week to discuss work (mean)	1.9	2.2	0.08	2.0	2.0	0.89
Average times asked opposite-cadre FLW to conduct visit (because you could not) in the past 30 days (mean)	1.3	1.6	0.19	1.4	1.7	0.39
Average times opposite-cadre FLW asked to conduct visit (because she could not) in the past 30 days (mean)	1.0	1.3	0.18	1.0	1.6	0.01

*Survey-weighted percentages and counts are reported to account for the survey design. Survey regression models were performed that accounted for village as the primary sampling unit and subcentre as the primary stratum within the sampling unit, and with proportional sampling weights at the FLW level. Survey logistic regression models were conducted for binary outcomes and survey Poisson regressions for count variables. Separate regression models were conducted for each outcome, for each cadre (AWW, ASHA); p values reflect comparisons of the intervention and control groups for each cadre.

†Three AWWs had missing responses for this outcome.

Table 3 describes the types of advice related to the team-based goals that maternal respondents from the postimplementation survey reported they received from FLWs. Mothers from intervention villages were significantly more likely than mothers from control villages to report that they received advice on iron-folic acid (IFA) tablets (goal 2), exclusive breast feeding (goal 3), keeping the cord clean (goal 4), starting complementary feeding at age 6 months (among women with infants 6–11 months old) (goal 5) and family planning among women with infants <6 months old (goal 6); differences between the two groups of women ranged from 8 (cord care) to 14 (family planning) percentage points. Receipt of advice about immediate breast feeding (goal 3) was not significantly different in intervention than control mothers. The overall percentage receiving the various types of advice varied, with complementary feeding and family planning advice being the least frequently reported. We also examined the per cent of women who received advice about specific health behaviours, stratified based on whether they received FLW home visits that were likely to have included that type of advice (online supplementary table 1). Frequencies of advice about behaviours tended to be much higher among women who received relevant home visits. In addition, significant differences between mothers from control and intervention villages in the frequencies of advice related to behaviours tended to be restricted to mothers who received home visits.

10.1136/bmjgh-2018-001146.supp2 Supplementary data

**Table 3:** Comparison of the percentage of maternal respondents from control and intervention villages who received different types of advice from front-line workers after implementation, as reported by maternal respondents in the Team-Based Goals and Incentives intervention trial in Begusarai, Bihar, 2012–2014*

Advice received during pregnancy from front-line worker	Team-based goal number †	No in model	Control (%) n=831	Intervention (%) n=859	P value
Advice on iron-folic acid tablets	2	1690	29.3	42.1	<0.01
**Advice received after delivery**					
Advice on immediate breast feeding	3	1690	33.1	38.4	0.25
Advice on exclusive breast feeding	3	1676	39.2	50.8	0.02
Advice on keeping cord clean	4	1690	32.7	41.4	0.03
Advice to start complementary feeding at age 6 months, among women with infant 6–11 months old	5	743	18.7	32.9	<0.01
Advice to start family planning at age 6 months, among women with infant <6 months old	6	896	12.4	22.6	0.01
Advice to start family planning at age 6 months, among women with infant 6–11 months old	6	748	20.7	22.6	0.72

*Survey-weighted percentages and counts are reported to account for the survey design. A separate logistic regression model was conducted for each outcome. Each model accounted for village as the primary sampling unit and subcentre as the primary stratum within the sampling unit, and with proportional sampling weights at the maternal respondent/household level; each model was also adjusted for maternal age (as a continuous variable) and caste (non-Hindu, Hindu scheduled caste/scheduled tribe (SC/ST), Hindu not SC/ST). P values reflect comparisons of the intervention and control groups from these models.

†See box 1 for goal to which this advice was related; information was not collected for goal 1 or 7.

Maternal respondents reported whether they received different types of home visits from FLWs, at baseline and postimplementation (table 4). There was a 15 absolute percentage point increase in antenatal home visits attributable to the TBGI intervention (p=0.03 for the interaction term). Home visits (>1 visit) within 24 hours after delivery (among women who had home births) were only assessed at postimplementation and were similar for both groups of women. Postnatal home visits (>1 visit) within 1 week of delivery and postpartum visits that addressed complementary feeding increased substantially from baseline to postimplementation in both intervention and control villages, but differences in these increases were not significantly different (per the DID analysis). There were also significantly more postpartum home visits that addressed family planning in the intervention group compared with the control group after implementation, but changes from baseline to postimplementation were not statistically significant.

**Table 4:** Impacts of the Team-Based Goals and Incentives (TBGI) intervention on home visits from front-line workers, as reported by maternal respondents, Begusarai, Bihar, 2012–2014*

Modelled indicators	Baseline (%)† (May to June 2012) n=1680				Postimplementation (%)† (November to December 2014) n=1690				Difference in difference‡ n=3370		
	No. in model	Control (n=849)	Intervention (n=831)	P value	No. in model	Control (n=831)	Intervention (n=859)	P value	No. in model	Per cent difference attributable to TBGI	P value
At least two antenatal home visits in final trimester	1664	38.2	33.3	0.33	1690	55.1	64.7	0.01	3354	14.5	0.027
At least one home visit within 24 hours of delivery, among women who had a home delivery	–	–	–	–	276	21.9	21.3	0.71	–	–	–
At least one home visit within 1 week of delivery	1679	11.2	9.8	0.80	1678	46.5	52.7	0.19	3357	7.6	0.42
Complementary feeding home visit for women with infant 6–11 months old	796	1.1	2.2	0.39	748	23.8	35.2	0.05	1544	10.6	0.86
Postpartum family planning home visits for women with infant <6 months old	820	11.4	20.7	0.07	896	9.8	16.9	0.04	1716	−2.2	0.87
Postpartum family planning home visits for women with infant 6–11 months old	820	15.1	13.5	0.80	748	17.0	18.8	0.75	1568	3.3	0.53

*Survey-weighted percentages and counts are reported to account for the survey design. Survey logistic regression models were performed that accounted for village as the primary sampling unit and subcentre as the primary stratum within the sampling unit, with proportional sampling weights at the maternal respondent/household level; each model also included maternal age (as a continuous variable) and caste (non-Hindu, Hindu scheduled caste/scheduled tribe (SC/ST), Hindu not SC/ST).

†Separate regression models were conducted for each outcome, at baseline and postimplementation; p values reflect comparisons of the intervention and control groups at each time point.

‡In order to estimate the effect of the TBGI intervention on a particular indicator, we conducted a separate regression model for each outcome that included all maternal respondents. These models each contained a term representing time (baseline or postimplementation), a term representing treatment (intervention or control) and an interaction of these two terms, which is represented by the difference-in-difference (DID) estimator and its p value. The DID reflects treatment effects (positive values reflect the amount of improvement attributable to the intervention).

Assessment of changes in behaviours related to goals of the TBGI intervention, as reported by maternal respondents, showed that the intervention resulted in 7 percentage points higher consumption of IFA tablets among women from intervention than control villages after implementation (table 5); however, overall, the receipt of IFA tablets declined from the preimplementation to postimplementation period. We also examined the frequency of these behaviours, stratified based on whether or not a respondent reported receiving relevant FLW home visits (online supplementary table 2). Frequencies of the goal-reported behaviours tended to be higher among women who had received FLW visits relevant to the behaviour.

10.1136/bmjgh-2018-001146.supp3 Supplementary data

**Table 5:** Impact of the Team-Based Goals and Incentives (TBGI) intervention on goal-related behaviours, as reported by maternal respondents, Begusarai, Bihar, 2012–2014*

Goal-related behaviours†	Goal number†	Baseline (%)‡ (May to June 2012) n=1680				Postimplementation (%)‡ (November to December 2014) n=1690				Difference in Difference§ n=3370		
		No in model	Control (n=849)	Intervention (n=831)	P value	No. in model	Control (n=831)	Intervention (n=859)	P value	No. in model	Per cent difference attributable to TBGI	P value
Obtained phone number for delivery	1											
A. Front-line worker’s (FLW) number		n.a.	n.a.	n.a.		1690	53.9	49.2	0.21	n.a.	n.a.	n.a.
B. Number for private vehicle		n.a.	n.a.	n.a.		1690	9.8	12.7	0.42	n.a.	n.a.	n.a.
C. Number for ambulance		n.a.	n.a.	n.a.		1690	13.2	15.8	0.19	n.a.		
D. Any of the above		n.a.	n.a.	n.a.		1690	58.7	55.4	0.39	n.a.	n.a.	n.a.
Received 90 IFA tablets	2	1678	25.0	23.1	0.51	1686	11.0	16.0	0.02	3364	6.8	0.02
Immediate breast feeding (within 1 hour of delivery)	3	1679	48.2	47.4	0.87	1687	54.2	60.3	0.19	3366	6.9	0.31
Nothing applied to the cord after cutting	4	1679	40.1	41.8	0.79	1559	53.2	53.2	0.98	3238	−1.7	0.80
Infant aged 6–11 months old ate cereal-based meal in previous day	5	815	40.1	45.3	0.35	744	54.3	69.4	<0.01	1559	9.8	0.14
Current use of any modern method of contraception	6											
A. Among women with infants <6 months old		818	17.2	19.0	0.71	892	10.5	10.3	0.69	1710	−1.6	0.73
B. Among women with infants 6–11 months old		811	23.6	17.5	0.13	726	18.9	18.0	0.71	1547	5.3	0.42
Child aged 6–11 months old received DPT3 vaccination¶	7	810	47.4	58.9	0.01	748	78.6	85.6	0.03	1558	−5.1	0.98

*Survey-weighted percentages and counts are reported to account for the survey design. Survey logistic regression models were performed that accounted for village as the primary sampling unit and subcentre as the primary stratum within the sampling unit, with proportional sampling weights at the maternal respondent/household level; each model also included maternal age (as a continuous variable) and caste (non-Hindu, Hindu scheduled caste/scheduled tribe (SC/ST), Hindu not SC/ST).

†See box 1 for further description of the goals.

‡Separate regression models were conducted for each outcome, at baseline and postimplementation; p values reflect comparisons of the intervention and control groups at each time point.

§In order to estimate the effect of the TBGI intervention on a particular indicator, we conducted a separate regression model for each outcome that included all maternal respondents. These models each contained a term representing time (baseline or postimplementation), a term representing treatment (intervention or control) and an interaction of these two terms, which is represented by the difference-in-difference (DID) estimator and its p value. The DID reflects treatment effects (positive values reflect the amount of improvement attributable to the intervention).

¶Vaccination was reported based on immunisation card or self-report.

DPT3, diphtheria, pertussis and tetanus; IFA, iron-folic acid; n.a., not applicable.

We also provide results for additional behaviours that were not directly related to the specific TBGI goals (online supplementary table 3). Several of these behaviours were positively impacted by the intervention (DID, p<0.10), including antenatal tetanus injections, IFA tablet consumption, exclusive breast feeding (among infants <6 months old), consumption of semisolid food (among infants >6 months old) and measles vaccine (among infants 9–11 months old).

10.1136/bmjgh-2018-001146.supp4 Supplementary data

## Discussion

This study evaluated the impact of a unique intervention that used team-based goals and non-monetary incentives to improve FLW effectiveness and motivation, and in turn, maternal RMNCHN-related behaviours. The intervention resulted in improvements in subcentre meeting attendance and in attitudes towards teamwork, especially with same-village ANMs, and in coordination of FLW planning. In addition, there were significantly higher frequencies of FLW antenatal home visits with women from intervention than control villages. Of note, complementary feeding visits were almost non-existent before the intervention, and were significantly higher in intervention than control areas after implementation, but the DID was not significant. Visits related to family planning remained low, likely reflecting the particular difficulties regarding this topic. In fact, 81% of AWWs and 76% of ASHAs reported that this was the hardest goal to achieve (data not shown). The intervention had the most consistent effect in increasing provision of health-related advice by ASHAs and AWWs to mothers. After accounting for baseline differences between women from the intervention and control villages, analyses indicated that the intervention resulted in significantly better performance on one RMNCHN goal—receipt of IFA tablets—but did not result in significantly better performance on other health behaviours related to the goals of the trial; it is also noteworthy that IFA receipt declined overall, which was likely a supply issue. We did observe improvements in several behaviours that were related to *Ananya* but not directly part of the TBGI goals, suggesting that the intervention had benefits beyond just the specific TBGI goals, and may have augmented the successes of the *Ananya* programme.

Some potential modifications of the study context and design and the implementation approach may have resulted in greater impacts on health-related behaviours. The intervention was implemented in the context of *Ananya*, and it is possible that it would have been more impactful as an isolated intervention, although testing it in the context of *Ananya* interventions is arguably more programmatically relevant. Obstacles to actual behaviour change may have included social norms (eg, related to feeding and family planning), supply chain factors (eg, for IFA, contraceptives), structural challenges (eg, ASHAs and AWWs were part of different government ministries), the effectiveness of FLW communications about the behaviours they were trying to change and development of the skills of beneficiaries necessary to adopt new behaviours (eg, breast feeding, complementary feeding).^4^ Insufficient focus on and accountability for behaviour change at the household level within the government’s primary healthcare system may have also been an overarching limitation to impact of the study. Moreover, the ability of the particular household items (eg, utensils, cookware) to motivate behaviour in this context is uncertain. Monetary incentives were considered, but it was suggested that the programme may have a greater chance of scale-up and sustainability if incentives were non-monetary and bulk discounts could reduce their cost. The targets for each behaviour tended to be far higher than the levels reported in our maternal surveys, so they may have been unrealistic. Although the goals were clearly assessed on a team basis (ie, they were collectively assessed for all FLWs attending the same health subcentre), few FLWs considered the FLWs who were not from their village to be part of their team. This suggests that some aspect of the intended ‘team’ affiliation may not have taken hold, especially as a health subcentre had not been structured systemically as a common catchment area for the village-based ASHAs and AWWs.

We are unaware of prior interventions that are directly comparable to the TBGI intervention, which is not surprising given its unique combination of teamwork, goal setting, multiple goals and non-monetary incentives. However, other studies do have comparable elements, and learnings from the TBGI intervention may have broader applicability. One prior study reported benefits of implementing a team structure among FLWs in Mozambique.^9^ Incentives used in other studies are varied, ranging from money to respect and recognition. Many studies have examined the motivational potential of various types of incentives, with highly variable impacts.^3^ Factors such as training, supervision, the health system structure and social context were also found to impact FLW motivation and performance^2^ and in turn, the potential impact of a particular incentive scheme. Of note, our study’s findings may be relevant to understanding performance-based financing (eg, pay for performance), a popular approach to incentivising improved quality of care through monetary means. Information regarding how and why it works (or does not work, depending on the context) is limited,^17^ and results have been somewhat mixed and controversial.^8^ It is often facility based and focused on operating budgets rather than teams or individuals. Our study suggests that non-monetary incentives and promotion of team-based performance may hold promise as an effective mechanism for improving healthcare worker effectiveness and, ultimately, health outcomes; in particular, it is possible that the goal-setting and team-building components could augment the effectiveness of performance-based financing.

Strengths of this study included the cluster randomised trial design; availability of survey data from the two main cadres of FLWs as well as from mothers, at baseline and postimplementation; and a carefully designed intervention that incorporated known concepts about motivation, goal setting and teamwork and consideration of the unique social context.^18^ Collection of baseline information was particularly important, given that the intervention took place in the context of the *Ananya* programme, which was expected to impact the same RMNCHN-related behaviours as the TBGI intervention. We used a DID approach for analyses, which we consider a strength in enabling attribution of change to the intervention; it also seemed appropriate, given that the study design incorporated randomisation at the cluster (health subcentre) rather than individual level, and the intervention was evaluated using independent cross-sectional surveys that were conducted before and after implementation. However, we also acknowledge that this analytic approach, because it incorporates an interaction, has less ability to achieve statistically significant results compared with a post-only comparison, and this might have resulted in some programmatically meaningful changes being not statistically significant (the power calculation used to design the original study was not based on a DID approach).^19^ To explore the robustness of the findings, we performed additional analyses using generalised estimating equations and mixed effects regression; these did not lead to any substantive differences in our main results (data not shown). The DID approach relies on the assumption that parallel trends existed in the tested outcomes in the control and intervention catchment areas before the programme was implemented; we only have data from two points in time (preimplementation and postimplementation) and are unable to confirm or refute whether this assumption was met. However, given the cluster randomised design, across many villages, we believe that the likelihood that non-parallel trends existed and would have driven our results is low.

The study also had some important limitations. Data on teamwork reflected attitudes only while data on team performance were not available. An ability to link team performance data with information from FLWs and maternal respondents would have been more informative for the evaluation of intervention impact. In addition, the evaluation survey mostly focused on discrete quantitative questions; more nuanced questions may have been informative, especially regarding changes in health behaviours (eg, receipt of less than 90 IFA tablets, or questions related to birth spacing). Data regarding ANMs were scanty, despite their being an important group of FLWs as supervisors and leaders of subcentre meetings. Information on DPT3 immunisation was usually from self-report rather than immunisation cards (95% vs 5%). Within this low literacy population, the ability to recall immunisation information is uncertain; however, we expect this error to be non-differential by intervention status. We did not employ any adjustment for multiple hypothesis testing; therefore, we cannot exclude the possibility that some results were significant by chance.

In summary, the TBGI intervention appeared to motivate improvements in FLW coordination and teamwork, especially of AWWs and ASHAs with ANMs. It increased FLW antenatal home visits and advice related to multiple domains of RMNCHN and impacted several maternal health-related behaviours. These results suggest that the approach of combining an integrated set of team-based coverage goals and targets, small non-cash incentives for teams who meet targets and other elements to motivate FLWs has promise for improving FLW motivation and performance, and FLW–beneficiary interactions. In alignment with this point, a prior study reported that various markers of intrinsic motivation (eg, empowerment, job satisfaction) were higher among FLWs from the intervention than control areas, as assessed after intervention.^20^ These types of improvements represent a first step towards improving health behaviours and outcomes but require further prospective, long-term, mixed methods investigations in other contexts.

Improving performance of primary care systems in low/middle-income countries (LMIC) is a major aspiration in the age of Universal Health Coverage and the Sustainable Development Goals. Major gaps in evidence have been identified in the area of performance measurement and management systems in LMIC.^21^ Our findings suggest that team-based incentives are a promising intervention to improve FLW performance in LMIC. However, improvements in study design are needed, particularly to increase policy relevance. Theory-based programme evaluations such as realist evaluation^22^ can contribute to increasing policy relevance by addressing the question ‘What works; how; why; and, who benefits.’ Future designs should also address the question of how would team-based incentives sustain FLW performance through time, once external support ends.^23^
