# Supplementary material for: Effects of team-based goals and non-monetary incentives on front-line health worker performance and maternal health behaviours: a cluster randomised controlled trial in Bihar, India
Source: BMJ Glob Health. 2019 Aug 26;4(4):e001146. doi: 10.1136/bmjgh-2018-001146 (PMC6730593; doi:10.1136/bmjgh-2018-001146)
Supplement: Supplementary data [file bmjgh-2018-001146supp001.pdf]

Statistical Methods for: **Effects of team-based goals and non-monetary incentives on frontline health worker performance and maternal health behaviours: a cluster randomized controlled trial in Bihar, India**

Table 2.

Demographics characteristics of FLW (ANMs, ASHAs and AWWs) as well as maternal respondents were compared for all MPR TBGI surveys by the baseline and post-intervention group (Table 1). Percentages were reported as crude percentages without adjusting for any survey design or weights. P value was calculated using two-sample t-test for the continuous variables and chi-square test for the categorical variables. If normality assumption was invalidated, Mann-Whitney U-Test would be performed for continuous variables instead. Fisher-exact test would be conducted for categorical variables with small numbers (<5) in any sub-group.

Table 3.

For all the outcomes in regarding to team-based goals in this table, we set up our survey design using village as primary sampling unit, sub-center as stratum with sampling weights at the household level. Our analytical cohort in this table was responses from the survey data for the AWW and ASHA at post-implementation. For the binary outcomes, rates of occurrence were reported by FLW group (AWW vs ASHA) and treatment groups (treatment vs control) using svytable command accounting for the survey design. To compare the difference of the responses between treatment and control group in both AWW and ASHA, survey logistic regressions were performed to get the p value for the difference in odds comparing the treatment vs the control without adjusting for any participant-level covariates. For discrete counts such as 'Times met with ANM outside subcenter meetings in past three months', survey Poisson regressions were conducted in a similar fashion via the same survey design set-up as survey logistic regressions. Since we had subcenter/stratum with one PSU in our data, we applied the 'options(survey.lonely.psu="adjust")' available in the 'survey' package in order to obtain stable estimates in all our regressions. All analysis was performed in R 3.4.3 via 'survey' package[1,2] available in R CRAN[3].

Table 5, 6

For all the binary outcomes in regarding to home visits from frontline workers (Table 5) and team-based goals (Table 6) as reported by maternal respondents in this table, we set up our survey design using village as primary sampling unit (psu), sub-center as stratum with sampling weights at the household level. Our analytical cohort in this table was maternal responses from the survey data in both baseline and post-implementation. Rates of occurrence of all our binary outcomes were reported by study period (baseline vs post-implementation) and treatment groups (treatment vs control) using svytable command accounting for the survey design. To compare the difference of the responses between treatment and control group in both baseline and post-implementation, survey logistic regressions were performed to

get the p value for the difference in odds of the outcome comparing the treatment vs the control adjusting for selected maternal respondents' demographic variables that were statistically significant. For each of our binary outcome, we also fit a separate survey logistic regression with all our baseline and post-implementation data to get the p value of the difference in difference estimator (DID, interaction between study period and treatment group), adjusting for selected maternal respondents' demographic variables that were statistically significant in Table 1. Specifically, the independent variables in the logistic regression for DID included treatment group, study period and the interaction of the two and selected demographic variables of the maternal respondents, while the dependent variable was our binary outcome of interest. To calculate percent difference between post-implementation and baseline in terms of difference of rates in the treatment vs control (also interpreted as the 'treatment effect'), we predicted the probability of outcome via our logistic regression with the interaction term and computed the difference in difference in probabilities of the outcome between the treatment and control group between post-implementation and baseline. Since we had subcenter/stratum with one PSU in our data, we applied the 'options(survey.lonely.psu="adjust")' available in the 'survey' package in order to obtain stable estimates in all our regressions. # of participants in each regression model was also reported in the tables. All analysis was performed in R 3.4.3 via 'survey' package[1,2] available in R CRAN[3].

Reference:

1. T. Lumley (2017) "survey: analysis of complex survey samples". R package version 3.32.

2. T. Lumley (2004) Analysis of complex survey samples. Journal of Statistical Software 9(1): 1-19

3. https://cran.rproject.org/web/packages/survey/index.html

Appendix. List of Variables for reproducibility

1. ANM

**Original Variable**	**Any Modification (R Codes)**	**Description**
t0_a1, t2_a1	data_anm_anly$live_svilage<-ifelse(data_anm_anly$endline==1, as.character(data_anm_anly$t2_a1), as.character(data_anm_anly$t0_a1))	Live in the village that provided service (y/n)
t2_a7, t0_a7	data_anm_anly$hindu<-ifelse(data_anm_anly$endline==1, as.character(data_anm_anly$t2_a7), as.character(data_anm_anly$t0_a7))	Hindu (y/n)
t2_a9, t0_a9	data_anm_anly$caste_hindu<-ifelse(data_anm_anly$endline==1 & data_anm_anly$hindu=="yes", as.character(data_anm_anly$t2_a9), ifelse(data_anm_anly$endline==0&	Caste (for Hindu Only)<br>- "Scheduled caste/tribe (SC/ST) (lowest caste)",

	data_anm_anly$hindu=="yes", as.character(data_anm_anly$t0_a9),NA)) data_anm_anly$caste_hindu<-ifelse(data_anm_anly$caste_hindu %in% c("scheduled caste", "scheduled tribe"),0, ifelse(data_anm_anly$caste_hindu %in% c("other backward caste"),1, ifelse(data_anm_anly$caste_hindu %in% c("general caste", "general caste"),2,NA))) data_anm_anly$caste_hindu<-factor(data_anm_anly$caste_hindu, levels=c(0,1,2), labels=c("Scheduled caste/tribe (SC/ST) (lowest caste)", "Other backward caste (socially and educationally disadvantaged)", "General Caste"))	- "Other backward caste (socially and educationally disadvantaged)"<br>- "General Caste"
t2_a11, t0_a11	data_anm_anly$college<-ifelse(data_anm_anly$endline==1, as.character(data_anm_anly$t2_a11), ifelse(data_anm_anly$endline==0, as.character(data_anm_anly$t0_a11),NA))	College (y/n)
treatment	data_anm_anly$trt<-ifelse(data_anm_anly$treatment=="control sub-centre",0,1)	Treatment group (y/n)
t2_a6, t0_a6	data_anm_anly$age<-ifelse(data_anm_anly$endline==1, data_anm_anly$t2_a6,data_anm_anly$t0_a6)	Age (continuous)
t2_a3, t0_a3	data_anm_anly$distance_sub<-ifelse(data_anm_anly$endline==1, data_anm_anly$t2_a3,data_anm_anly$t0_a3)	Distance from subcenter village (continuous)

2. ASHA/AWW

**Original Variable**	**Any Modification (R Codes)**	**Description**
asha		Asha (y/n)<br>-0: AWW<br>-1: ASHA
t0_a1, t2_a1	data_asha_aww_anly$live_svilage<-ifelse(data_asha_aww_anly$endline==1, as.character(data_asha_aww_anly$t2_a1), as.character(data_asha_aww_anly$t0_a1))	Live in the village that provided service (y/n)
t2_a7, t0_a7	data_asha_aww_anly$hindu<-ifelse(data_asha_aww_anly$endline==1,	Hindu (y/n)

	as.character(data_asha_aww_anly$t2_a7),<br>as.character(data_asha_aww_anly$t0_a7))	
t2_a9, t0_a9	data_asha_aww_anly$caste_hindu<-ifelse(<br>data_asha_aww_anly$endline==1 &<br>data_asha_aww_anly$hindu=="yes",<br>as.character(data_asha_aww_anly$t2_a9),<br>ifelse(data_asha_aww_anly$endline==0&<br>data_asha_aww_anly$hindu=="yes",<br>as.character(data_asha_aww_anly$t0_a9),<br>NA))<br>data_asha_aww_anly$caste_hindu<-ifelse(<br>data_asha_aww_anly$caste_hindu %in%<br>c("scheduled caste",<br>"scheduled tribe"),0,<br>ifelse(data_asha_aww_anly$caste_hindu %in%<br>c("other backward caste"),1,<br>ifelse(data_asha_aww_anly$caste_hindu %in%<br>c("general caste", "general caste"),2,NA)))<br>data_asha_aww_anly$caste_hindu<-factor(data_asha_aww_anly$caste_hindu,<br>levels=c(0,1,2),<br>labels=c("Scheduled caste/tribe (SC/ST) (lowest caste)",<br>"Other backward caste (socially and educationally disadvantaged)",<br>"General Caste"))	Caste (for Hindu Only)<br>- "Scheduled caste/tribe (SC/ST) (lowest caste)",<br>- "Other backward caste (socially and educationally disadvantaged)"<br>- "General Caste"
treatment	data_asha_aww_anly$trt<-ifelse(data_asha_aww_anly$treatment=="control sub-centre",0,1)	Treatment group (y/n)
t2_a6, t0_a6	data_asha_aww_anly$age<-ifelse(data_asha_aww_anly$endline==1,<br>data_asha_aww_anly$t2_a6,data_asha_aww_anly$t0_a6)	Age (continuous)
t2_flw2_attscm_3more		Attended three or more subcenter meetings in the past three months (y/n)
t2_flw2_team_othflw		Consider Other FLW of the village (other cadre) part of the team (y/n)
t2_flw2_team_anm		Consider Subcenter Auxiliary Nurse Midwife (ANM) part of the team (y/n)

t2_flw2_team_sameflw_sc		Consider Same-cadre frontline workers (FLWs) at subcenter part of the team (y/n)
t2_flw2_team_othflw_sc		Consider Other-cadre FLWs at the subcenter part of the team (y/n)
t2_flw2_teamhelp_always		Always expected to plan with team (y/n)
t2_flw2_teamplan_always		Always expected to plan with team
t2_flw2_teammeet_always		Always expected to meet regularly with team
t2_flw2_anmjointvis		Any joint visits with ANM in past month
t2_asha_wt, t2_aww_wt	data_asha_aww_endline$t2_wt<-ifelse(data_asha_aww_endline$asha==1, data_asha_aww_endline$t2_asha_wt, data_asha_aww_endline$t2_aww_wt)	Endline household weight
village		Village ID
sc_code		Subcenter ID
t2_flw2_meetanm_out sidesc		Times met with ANM outside subcenter meetings in past three months (number)
t2_flw2_numjointvisit		Number of joint home visits in the past week (number)
t2_flw2_meet_othflw		Number of times met with opposite-cadre FLW in past week to discuss work (number)
t2_flw2_othflw_cover		Number of times asked opposite-cadre FLW to conduct visit (because you could not) in past 30 days (number)
t2_flw2_you_cover		Number of times opposite-cadre FLW asked to conduct visit (because she could not) in past 30 days (number)

3. Maternal Respondents (or household survey)

**Original Variable**	**Any Modification (R Codes)**	**Description**
t2_hindu, t0_hindu	data_hh_anly$hindu<-ifelse(data_hh_anly$endline==1, data_hh_anly$t2_hindu,data_hh_anly$t0_hindu)	Hindu (y/n)

	data_hh_anly$hindu<-<br>ifelse(data_hh_anly$hindu==1,"yes",<br>ifelse(data_hh_anly$hindu==0,"no",NA))	
t2_scst, t0_scst	data_hh_anly$caste_hindu<-<br>ifelse(data_hh_anly$endline==1 &<br>data_hh_anly$hindu=="yes" &<br>is.na(data_hh_anly$hindu)==F ,<br>data_hh_anly$t2_scst,ifelse(data_hh_anly$endline==0&<br>data_hh_anly$hindu=="yes" &<br>is.na(data_hh_anly$hindu)==F,data_hh_anly$t0_scst,NA))<br>data_hh_anly$caste_hindu<-<br>factor(data_hh_anly$caste_hindu,levels=c(0,1),labels=c("No<br>t Scheduled caste/tribe (SC/ST)","Scheduled caste/tribe<br>(SC/ST)"))	Caste (for Hindu Only)<br>- "Not Scheduled caste/tribe (SC/ST)"<br>- "Scheduled caste/tribe (SC/ST)"
treatment		Treatment group (y/n)
t2_agefml_15to19,<br>t0_agefml_15to19,<br>t2_agefml_20to24,<br>t0_agefml_20to24,<br>t2_agefml_25to29,<br>t0_agefml_25to29,<br>t2_agefml_30to34,<br>t0_agefml_30to34,<br>t2_agefml_35older,<br>t0_agefml_35older	data_hh_anly$hh_age_15to19<-<br>ifelse(data_hh_anly$endline==1,<br>data_hh_anly$t2_agefml_15to19,<br>data_hh_anly$t0_agefml_15to19)<br>data_hh_anly$hh_age_20to24<-<br>ifelse(data_hh_anly$endline==1,<br>data_hh_anly$t2_agefml_20to24,<br>data_hh_anly$t0_agefml_20to24)<br>data_hh_anly$hh_age_25to29<-<br>ifelse(data_hh_anly$endline==1,<br>data_hh_anly$t2_agefml_25to29,<br>data_hh_anly$t0_agefml_25to29)<br>data_hh_anly$hh_age_30to34<-<br>ifelse(data_hh_anly$endline==1,<br>data_hh_anly$t2_agefml_30to34,<br>data_hh_anly$t0_agefml_30to34)<br>data_hh_anly$hh_age_35older<-<br>ifelse(data_hh_anly$endline==1,data_hh_anly$t2_agefml_3<br>5older,<br>data_hh_anly$t0_agefml_35older)<br>data_hh_anly$hh_age_cat<-<br>ifelse(data_hh_anly$hh_age_15to19==1 &<br>is.na(data_hh_anly$hh_age_15to19)==F,1,<br>ifelse(data_hh_anly$hh_age_20to24==1 &<br>is.na(data_hh_anly$hh_age_20to24)==F,2,<br>ifelse(data_hh_anly$hh_age_25to29==1 &<br>is.na(data_hh_anly$hh_age_25to29)==F,3,<br>ifelse(data_hh_anly$hh_age_30to34==1 &<br>is.na(data_hh_anly$hh_age_30to34)==F,4,<br>ifelse(data_hh_anly$hh_age_35older==1 &<br>is.na(data_hh_anly$hh_age_35older)==F,5,NA)))))<br>data_hh_anly$hh_age_cat<-<br>factor(data_hh_anly$hh_age_cat,levels=c(1:5),labels=c<br>("15-19","20-24","25-29","30-34","35-49"))	Age Categorical-<br>- 15 to 19<br>- 20 to 24<br>- 25 to 29<br>- 30 to 34<br>- 35 or older

t2_d01,<br>t0_d01	data_hh_anly$hh_numkids_cat<-ifelse(data_hh_anly$endline==1,data_hh_anly$t2_d01,data_hh_anly$t0_d01)<br>data_hh_anly$hh_numkids_cat<-ifelse(data_hh_anly$hh_numkids_cat<4& is.na(data_hh_anly$hh_numkids_cat)==F,data_hh_anly$hh_numkids_cat, ifelse(data_hh_anly$hh_numkids_cat>=4& is.na(data_hh_anly$hh_numkids_cat)==F,4,NA))<br>data_hh_anly$hh_numkids_cat<-factor(data_hh_anly$hh_numkids_cat,levels=1:4,labels=c("1 Child","2 Children","3 Children","4 or More Children"))	Numbers of Kids<br>- "1 Child"<br>- "2 Children"<br>- "3 Children"<br>- "4 or More Children"
t2_literate,<br>t0_literate	data_hh_anly$hh_literate<-ifelse(data_hh_anly$endline==1,data_hh_anly$t2_literate,data_hh_anly$t0_literate)	Literacy (y/n)
t2_bpl,<br>t0_bpl	data_hh_anly$hh_bpl<-ifelse(data_hh_anly$endline==1,data_hh_anly$t2_bpl,data_hh_anly$t0_bpl)	BPL (y/n)
t2_wealthindex_qurt,<br>t0_wealthindex_qurt	data_hh_anly$hh_wealthindex_qurt<-ifelse(data_hh_anly$endline==1,data_hh_anly$t2_wealthindex_qurt,data_hh_anly$t0_wealthindex_qurt)	Wealth Quartile<br>- 1<br>- 2<br>- 3<br>- 4
t2_agefml,<br>t0_agefml	data_hh_anly$hh_age<-ifelse(data_hh_anly$endline==1,data_hh_anly$t2_agefml,data_hh_anly$t0_agefml)	Age (continuous)
t2_hhsize,<br>t0_hhsize	data_hh_anly$hh_size<-ifelse(data_hh_anly$endline==1,data_hh_anly$t2_hhsize,data_hh_anly$t0_hhsize)	Household Size (continuous)
t2_flw_visit_tot_3trim_atl2,<br>t0_flw_visit_tot_3trim_atl2	data_hh_anly$ flw_visit_tot_3trim_atl2<-ifelse(data_hh_anly$endline==1, data_hh_anly$ t2_flw_visit_tot_3trim_atl2, dat_hh_anly$ t0_flw_visit_tot_3trim_atl2)	At least antenatal home visits in the final trimester (y/n)
t2_flw_visit_firstmonth,<br>t0_flw_visit_firstmonth	data_hh_anly$ flw_visit_firstmonth <-ifelse(data_hh_anly$endline==1, data_hh_anly$ t2_ flw_visit_firstmonth, dat_hh_anly$ t0_flw_visit_firstmonth)	At least one home visit within 1 month of delivery (y/n)
t2_flw_visit_first24_homdel,<br>t0_flw_visit_first24_homdel	data_hh_anly$ flw_visit_first24_homdel <-ifelse(data_hh_anly$endline==1, data_hh_anly$ t2_ flw_visit_first24_homdel, dat_hh_anly$ t0_flw_visit_first24_homdel)	At least one home visit within 24 hour of home delivery (y/n)
t0_focal_child_agemths,<br>t2_focal_child_agemths,<br>t2_flw_visit_compfeed,<br>t0_flw_visit_compfeed	data_hh_anly$t0_compfeed_child6_11<-ifelse(data_hh_anly$baseline==1 & data_hh_anly$t0_focal_child_agemths>=6 & is.na(data_hh_anly$t0_focal_child_agemths)==F & data_hh_anly$t0_focal_child_agemths<=11,data_hh_anly$t0_flw_visit_compfeed,NA)<br><br>data_hh_anly$t2_compfeed_child6_11<-ifelse(data_hh_anly$baseline==0 & data_hh_anly$t2_focal_child_agemths>=6 &	Home Visit for Complementary Feeding for Women with infants 6-11 months (y/n)

	is.na(data_hh_anly$t2_focal_child_agemths)==F & data_hh_anly$t2_focal_child_agemths<=11,data_hh_anly$t2_flw_visit_compfeed,NA)<br><br>data_hh_anly $compfeed_child6_11<- ifelse(data_hh_anly$endline==1, data_hh_anly$ t2_compfeed_child6_11, dat_hh_anly$ t0_compfeed_child6_11	
t2_focal_child_agemths, t0_focal_child_agemths, t2_flw_visit_flyplan, t0_flw_visit_flyplan	data_hh_anly$t0_flyplan_childless6<- ifelse(data_hh_anly$baseline==1 & data_hh_anly$t0_focal_child_agemths<6& is.na(data_hh_anly$t0_focal_child_agemths)==F ,data_hh_anly$t0_flw_visit_flyplan,NA)<br><br>data_hh_anly$t2_flyplan_childless6<- ifelse(data_hh_anly$baseline==0 & data_hh_anly$t2_focal_child_agemths<6& is.na(data_hh_anly$t2_focal_child_agemths)==F ,data_hh_anly$t2_flw_visit_flyplan,NA)<br><br>data_hh_anly $ flyplan_childless6 <- ifelse(data_hh_anly$endline==1, data_hh_anly$ t2_flyplan_childless6, dat_hh_anly$ t0_flyplan_childless6	Postpartum family planning for women with infants less than 6 months (y/n)
t2_focal_child_agemths, t0_focal_child_agemths, t2_flw_visit_flyplan, t0_flw_visit_flyplan	data_hh_anly$t0_flyplan_child_6_11<- ifelse(data_hh_anly$baseline==1 &data_hh_anly$t0_focal_child_agemths<=11& data_hh_anly$t0_focal_child_agemths>=6& is.na(data_hh_anly$t0_focal_child_agemths)==F ,data_hh_anly$t0_flw_visit_flyplan,NA)<br><br>data_hh_anly$t2_flyplan_child_6_11<- ifelse(data_hh_anly$baseline==0 & data_hh_anly$t2_focal_child_agemths<=11 & data_hh_anly$t2_focal_child_agemths>=6& is.na(data_hh_anly$t2_focal_child_agemths)==F ,data_hh_anly$t2_flw_visit_flyplan,NA)<br><br>data_hh_anly $ flyplan_child_6_11<- ifelse(data_hh_anly$endline==1, data_hh_anly$ t2_flyplan_child_6_11, dat_hh_anly$ t0_flyplan_child_6_11	Postpartum family planning for women with infants 6-11 months (y/n)
t0_anc_transp_flwnum, t2_anc_transp_flwnum	data_hh_anly $ anc_transp_flwnum <- ifelse(data_hh_anly$endline==1, data_hh_anly$ t2_anc_transp_flwnum, dat_hh_anly$ t0_anc_transp_flwnum	Obtain Number of Frontier workers' phone (y/n)
t0_anc_transp_pvtvehnum, t2_anc_transp_pvtvehnum	data_hh_anly $ anc_transp_pvtvehnum <- ifelse(data_hh_anly$endline==1, data_hh_anly$ t2_anc_transp_pvtvehnum, dat_hh_anly$ t0_anc_transp_pvtvehnum	Obtain Number of Private Vehicle (y/n)
t2_anc_transp_anynum,	data_hh_anly $ anc_transp_anynum <- ifelse(data_hh_anly$endline==1,	Obtain Number of the Ambulance (y/n)

t0_anc_transp_anynum	data_hh_anly$ t2_anc_transp_anynum,<br>dat_hh_anly$ t0_anc_transp_anynum	
t2_anc_transp_anynum,<br>t0_anc_transp_anynum	data_hh_anly $ anc_transp_anynum <-<br>ifelse(data_hh_anly$endline==1,<br>data_hh_anly$ t2_anc_transp_anynum,<br>dat_hh_anly$ t0_anc_transp_anynum	Obtain Any Number (y/n)
t2_anc_ifa_atl90rec,<br>t0_anc_ifa_atl90rec	data_hh_anly $ anc_ifa_atl90rec <-<br>ifelse(data_hh_anly$endline==1,<br>data_hh_anly$ t2_anc_ifa_atl90rec,<br>dat_hh_anly$ t0_anc_ifa_atl90rec	Received 90 IFA tablets (y/n)
t2_dppc_immbrfeed,<br>t0_dppc_immbrfeed	data_hh_anly $ dppc_immbrfeed <-<br>ifelse(data_hh_anly$endline==1,<br>data_hh_anly$ t2_dppc_immbrfeed ,<br>dat_hh_anly$ t0_dppc_immbrfeed	Immediate Breastfeeding (y/n)
t2_f38,<br>t0_f38	data_hh_anly$t0_cord_notapplied<-<br>abs(as.numeric(data_hh_anly$t0_f38)-2)<br><br>data_hh_anly$t2_cord_notapplied<-<br>abs(as.numeric(data_hh_anly$t2_f38)-2)<br><br>data_hh_anly $ cord_notapplied <-<br>ifelse(data_hh_anly$endline==1,<br>data_hh_anly$ t2_cord_notapplied,<br>dat_hh_anly$ t0_cord_notapplied	Nothing applied to cord after cutting
t2_focal_child_agemths,<br>t0_focal_child_agemths,<br>t2_feed_anycereal_prevday,<br>t0_feed_anycereal_prevday	data_hh_anly$t0_feed_anycereal_prevday_6_11<-<br>ifelse(data_hh_anly$baseline==1 &<br>data_hh_anly$t0_focal_child_agemths>=6 &<br>data_hh_anly$t0_focal_child_agemths<=11 &<br>is.na(data_hh_anly$t0_focal_child_agemths)==F,data_hh_anly$t0_feed_anycereal_prevday,NA)<br><br>data_hh_anly$t2_feed_anycereal_prevday_6_11<-<br>ifelse(data_hh_anly$baseline==0 &<br>data_hh_anly$t2_focal_child_agemths>=6 &<br>data_hh_anly$t2_focal_child_agemths<=11 &<br>is.na(data_hh_anly$t2_focal_child_agemths)==F,data_hh_anly$t2_feed_anycereal_prevday,NA)<br><br>data_hh_anly $ feed_anycereal_prevday_6_11<-<br>ifelse(data_hh_anly$endline==1,<br>data_hh_anly$ t2_feed_anycereal_prevday_6_11 ,<br>dat_hh_anly$ t0_feed_anycereal_prevday_6_11	Infant age 6-11 months old ate cereal-based meal in previous day (y/n)
t2_focal_child_agemths,<br>t0_focal_child_agemths,<br>t2_fp_contr_anymodern,<br>t0_fp_contr_anymodern	data_hh_anly$t0_fp_contr_anymodern_less6<-<br>ifelse(data_hh_anly$baseline==1 &<br>data_hh_anly$t0_focal_child_agemths<6 &<br>is.na(data_hh_anly$t0_focal_child_agemths)==F,data_hh_anly$t0_fp_contr_anymodern,NA)<br><br>data_hh_anly$t2_fp_contr_anymodern_less6<-<br>ifelse(data_hh_anly$baseline==0 &<br>data_hh_anly$t2_focal_child_agemths<6 &	Current use of any modern method of contraception (infants<6 months) (y/n)

	is.na(data_hh_anly$t2_focal_child_agemths)==F,data_hh_anly$t2_fp_contr_anymodern,NA)<br><br>data_hh_anly $ fp_contr_anymodern_less6<-ifelse(data_hh_anly$endline==1, data_hh_anly$ t2_fp_contr_anymodern_less6, dat_hh_anly$ t0_fp_contr_anymodern_less6	
t2_focal_child_agemths, t0_focal_child_agemths, t2_fp_contr_anymodern, t0_fp_contr_anymodern	data_hh_anly$t0_fp_contr_anymodern_6_11<-ifelse(data_hh_anly$baseline==1 & data_hh_anly$t0_focal_child_agemths>=6 & data_hh_anly$t0_focal_child_agemths<=11 & is.na(data_hh_anly$t0_focal_child_agemths)==F,data_hh_anly$t0_fp_contr_anymodern,NA)<br><br>data_hh_anly$t2_fp_contr_anymodern_6_11<-ifelse(data_hh_anly$baseline==0 & data_hh_anly$t2_focal_child_agemths>=6 & data_hh_anly$t2_focal_child_agemths<=11 & is.na(data_hh_anly$t2_focal_child_agemths)==F,data_hh_anly$t2_fp_contr_anymodern,NA)<br><br>data_hh_anly $ fp_contr_anymodern_6_11<-ifelse(data_hh_anly$endline==1, data_hh_anly$ t2_fp_contr_anymodern_6_11, dat_hh_anly$ t0_fp_contr_anymodern_6_11	Current use of any modern method of contraception (infants 6-11 months)
t2_imm_dpt3_gt6, t0_imm_dpt3_gt6m	data_hh_anly $ imm_dpt3_gt6<-ifelse(data_hh_anly$endline==1, data_hh_anly$ t2_imm_dpt3_gt6, dat_hh_anly$ t0_imm_dpt3_gt6	Child age 6-11 months old received DPT3 vaccination
t0_n22, t2_flw_visit_first24, t2_flw_visit_firstweek	data_hh_anly$t0_flw_firstweek_24hrinclude<-ifelse(data_hh_anly$t0_n22=="yes" & is.na(data_hh_anly$t0_n22)==F,1,ifelse(data_hh_anly$baseline==1 & (is.na(data_hh_anly$t0_n22)\|data_hh_anly$t0_n22=="no" & is.na(data_hh_anly$t0_n22)==F),0,NA))<br><br>data_hh_anly$t2_flw_firstweek_24hrinclude<-ifelse((data_hh_anly$t2_flw_visit_first24==1 & is.na(data_hh_anly$t2_flw_visit_first24)==F)|(data_hh_anly$t2_flw_visit_firstweek==1 & is.na(data_hh_anly$t2_flw_visit_firstweek)==F),1,ifelse(data_hh_anly$endline==1 & ((data_hh_anly$t2_flw_visit_first24==0 & is.na(data_hh_anly$t2_flw_visit_first24)==F) & (data_hh_anly$t2_flw_visit_firstweek==0 & is.na(data_hh_anly$t2_flw_visit_firstweek)==F)),0,NA))<br><br>data_hh_anly $ flw_firstweek_24hrinclude <-ifelse(data_hh_anly$endline==1, data_hh_anly$ t2_flw_firstweek_24hrinclude, dat_hh_anly$ t0_flw_firstweek_24hrinclude	At least one Home Visit in first week after delivery (y/n)
