# Supplementary material for: Effects of team-based goals and non-monetary incentives on front-line health worker performance and maternal health behaviours: a cluster randomised controlled trial in Bihar, India
Source: BMJ Glob Health. 2019 Aug 26;4(4):e001146. doi: 10.1136/bmjgh-2018-001146 (PMC6730593; doi:10.1136/bmjgh-2018-001146)
Supplement: Supplementary data [file bmjgh-2018-001146supp002.pdf]

Supplementary Table 1. Comparison of the percentage of maternal respondents from control and intervention villages who received different types of advice from frontline workers (FLW), stratified by types of FLW visits they received, as reported by maternal respondents post-implementation of the Team-Based Goals and Incentives intervention trial in Begusarai, Bihar, 2012-2014.[a]

Advice received from a FLW	Type of FLW visit received	N	Control (%)	N	Inter-vention (%)	p-value
Advice on iron-folic acid tablets	≥2 antenatal care (ANC) visits	446	37.9	570	52.1	0.03
	<2 ANC visits	385	18.8	289	23.7	0.09
Advice on immediate breastfeeding	≥2 ANC visits	446	52.1	570	55.7	0.52
	<2 ANC visits	385	9.7	289	6.7	0.43
	Home visit within 24 hours of delivery, among women who had a home delivery	32	37.3	37	68.8	0.01
	No home visit within 24 hours of delivery, among women who had a home delivery	107	8.6	100	11.4	0.55
Advice on exclusive breastfeeding	≥2 ANC visits	439	61.0	567	69.2	0.15
	>2 ANC visits	383	12.9	287	17.1	0.27
	Home visit within 24 hours of delivery, among women who had a home delivery	31	51.6	37	87.3	<0.01
	No home visit within 24 hours of delivery, among women who had a home delivery	106	14.6	99	17.1	0.61
	Home visit within 1 week of delivery	403	75.8	475	86.8	0.03
	No home visit within 1 week of delivery	417	8.0	372	11.9	0.12
Advice on keeping cord clean	≥2 ANC visits	446	52.1	570	59.1	0.15
	<2 ANC visits	385	9.0	289	9.1	0.80
	Home visit within 24 hours of delivery, among women who had a home delivery	32	55.8	37	73.2	0.25

Carmichael SL, *et al. BMJ Global Health* 2019; 4:e001146. doi: 10.1136/bmjgh-2018-001146

	No home visit within 24 hours of delivery, among women who had a home delivery	107	10.6	100	10.5	0.93
	Home visit within 1 week of delivery	408	63.9	479	71.3	0.07
	No home visit within 1 week of delivery	419	5.8	372	9.1	0.18
Advice to start complementary feeding at age 6 months, among women with infant 6-11 months old	Received complementary feeding home visit for women with infant 6-11 months old	107	83.4	140	93.0	<0.01
	Did not receive complementary feeding home visit for women with infant 6-11 months old	n.a.				
Advice to start family planning at age 6 months, among women with infant <6 months old	≥2 ANC visits	229	19.2	303	30.4	0.02
	<2 ANC visits	216	4.6	194	9.8	0.20
	Did not receive postpartum family planning home visits for women with infant <6 months old	n.a.				
	Received postpartum family planning home visits for women with infant <6 months old	396	2.8	410	6.4	0.04
Advice to start family planning at age 6 months, among women with infant 6-11 months old	Did not receive postpartum family planning home visits for women with infant 6-11 months old	n.a.				
	Received received postpartum family planning home visits for women with infant 6-11 months old	323	4.4	295	4.7	0.79

[a]Survey-weighted percentages and counts are reported, to account for the survey design. A separate logistic regression model was conducted for each outcome. Each model accounted for village as the primary sampling unit and sub-center as the primary stratum within the sampling unit, and with proportional sampling weights at the maternal respondent/household level; each model was also adjusted for maternal age (as a continuous variable) and caste (non-Hindu, Hindu SC/ST, Hindu not SC/ST).

n.a. = not available (these questions were not asked, due to skip patterns inherent to the questionnaires)
