# Supplementary material for: Effects of team-based goals and non-monetary incentives on front-line health worker performance and maternal health behaviours: a cluster randomised controlled trial in Bihar, India
Source: BMJ Glob Health. 2019 Aug 26;4(4):e001146. doi: 10.1136/bmjgh-2018-001146 (PMC6730593; doi:10.1136/bmjgh-2018-001146)
Supplement: Supplementary data [file bmjgh-2018-001146supp003.pdf]

Supplementary Table 2. Impact of the Team-Based Goals and Incentives (TBGI) intervention on goal-related behaviors, as reported by maternal respondents, Begusarai, Bihar, 2012-2014, stratified by whether respondents reported receiving relevant home visits from frontline workers.[a]

			Baseline (%)[c] (May-June 2012) n=1680				Post-implementation (%)[c] (Nov-Dec 2014) n=1690				Difference in Difference[d] n=3370		
Goal-related behaviors[b]	Goal number[b]	Stratified by	No. in model	Control (n=849)	Interven-tion (n=831)	p-value	No. in model	Control (n=831)	Interven-tion (n=859)	p-value	No. in model	Percent difference attributable to TBGI	p-value
Stratified based on receiving at least 2 antenatal home visits in the final trimester:													
Obtained phone number for delivery:													
a. Frontline worker's (FLW) number	1	Less than 2 visits	n.a.	n.a.	n.a.	n.a.	674	26.6	19.2	0.08	n.a.	n.a.	n.a.
		At least 2 visits	n.a.	n.a.	n.a.	n.a.	1016	66.4	67.3	0.76	n.a.	n.a.	n.a.
b. Number for private vehicle		Less than 2 visits	n.a.	n.a.	n.a.	n.a.	674	2.5	4.4	0.22	n.a.	n.a.	n.a.
		At least 2 visits	n.a.	n.a.	n.a.	n.a.	1016	14.7	30.5	<0.01	n.a.	n.a.	n.a.
c. Number for ambulance		Less than 2 visits	n.a.	n.a.	n.a.	n.a.	674	5.0	8.9	0.07	n.a.	n.a.	n.a.
		At least 2 visits	n.a.	n.a.	n.a.	n.a.	1016	16.5	28.8	<0.01	n.a.	n.a.	n.a.
Received 90 IFA tablets	2	Less than 2 visits	1086	25.1	21.5	0.37	673	7.9	11.9	0.02	1759	6.0	0.06
		At least 2 visits	578	25.7	27.4	0.71	1013	13.5	18.2	0.07	1591	5.6	0.25
Immediate breastfeeding (within 1 hour of delivery)	3	Less than 2 visits	1087	41.3	41.8	0.93	672	49.6	60.5	0.08	1759	8.2	0.18
		At least 2 visits	578	58.2	56.2	0.84	1015	58.0	60.2	0.78	1593	6.4	0.64

Carmichael SL, *et al. BMJ Global Health* 2019; 4:e001146. doi: 10.1136/bmjgh-2018-001146

Nothing applied to the cord after cutting	4	Less than 2 visits	1087	52.0	52.5	0.99	625	53.3	53.5	0.94	1712	-0.5	0.96
		At least 2 visits	578	18.8	19.0	1.00	934	53.2	53.1	0.96	1512	-0.8	0.99
Stratified based on complementary feeding home visit for women with infant 6-11 months old:												
Infant age 6-11 months old ate cereal-based meal in previous day	5	Did not receive complementary feeding home visit	782	41.5	55.1	0.47	519	52.8	65.8	0.02	1301	-4.7	0.200
		Received complementary feeding home visit	11	0	51.2	<0.001	225	59.4	75.9	0.01	236	-5.1	<0.001
Stratified based on postpartum family planning home visits for women with infant <6 months old:												
Current use of any modern method of contraception, among women with infants <6 months old	6	No Postpartum family planning home visits	712	11.7	10.0	0.53	762	7.8	8.3	0.89	1474	-0.35	0.666
		Had Postpartum family planning home visits	106	59.9	53.4	0.70	130	27.5	18.2	0.39	236	-1.2	0.816
Stratified based on postpartum family planning home visits for women with infant 6-11 months old												
Current use of any modern method of contraception, among women with infants	6	No Postpartum family planning home visits	721	16.8	13.7	0.33	598	15.7	14.7	0.56	1319	8.1	0.747
		Had Postpartum family	91	61.6	43.1	0.30	128	33.9	32.3	0.84	219	7.3	0.345

Carmichael SL, *et al. BMJ Global Health* 2019; 4:e001146. doi: 10.1136/bmjgh-2018-001146

6-11 months old*		planning home visits												

[a]Survey-weighted percentages and counts are reported, to account for the survey design. Survey logistic regression models were performed that accounted for village as the primary sampling unit and sub-center as the primary stratum within the sampling unit, with proportional sampling weights at the maternal respondent/household level; each model also included maternal age (as a continuous variable) and caste (non-Hindu, Hindu SC/ST, Hindu not SC/ST).

[b]See Table 1 for further description of the Goals.

[c]Separate regression models were conducted for each outcome, at baseline and post-implementation; p-values reflect comparisons of the intervention and control groups, at each time point.

[d]In order to estimate the effect of the TBGI intervention on a particular indicator, we conducted a separate regression model for each outcome that included all maternal respondents. These models each contained a term representing time (baseline or post-implementation), a term representing treatment (intervention or control), and an interaction of these two terms, which is represented by the difference in difference estimator (DID) and its p-value. The DID reflects treatment effects (positive values reflect the amount of improvement attributable to the intervention).

Carmichael SL, *et al. BMJ Global Health* 2019; 4:e001146. doi: 10.1136/bmjgh-2018-001146
