# Supplementary material for: Effects of team-based goals and non-monetary incentives on front-line health worker performance and maternal health behaviours: a cluster randomised controlled trial in Bihar, India
Source: BMJ Glob Health. 2019 Aug 26;4(4):e001146. doi: 10.1136/bmjgh-2018-001146 (PMC6730593; doi:10.1136/bmjgh-2018-001146)
Supplement: Supplementary data [file bmjgh-2018-001146supp004.pdf]

Supplementary Table 3. Impacts of the Team-Based Goals and Incentives (TBGI) intervention on selected behaviors, as reported by maternal respondents, Begusarai, Bihar, 2012-2014.[a]

Modeled indicators	Baseline (%)[b] (May-June 2012)			Post-implementation (%)[b] (Nov-Dec 2014)			Difference in Difference[c]	
	Control	Intervention	p-value	Control	Intervention	p-value	Percent difference attributable to TBGI	p-value
**Antenatal care**								
At least 2 TT injections or booster injection	94.5	93.1	0.46	83.8	89.5	0.02	7.1	0.08
At least 90 IFA tablets consumed	12.8	11.1	0.58	10.9	15.9	0.01	6.5	0.06
**Delivery and newborn care**								
Facility delivery	66.5	70.1	0.22	81.4	83.4	0.35	-1.6	0.88
Bath delayed by at least 2 days	44.9	48.0	0.48	46.1	46.1	0.95	-3.1	0.54
Skin-to-skin care	22.0	22.0	0.88	46.7	52.0	0.35	5.3	0.51
Exclusive breastfeeding for 6 months, reported for children 6 months or older (no water)	32.7	38.5	0.35	30.2	54.8	<0.01	18.5	0.03
Exclusive breastfeeding in past 24 hours, reported for children younger than 6 months[d]	51.3	43.1	0.10	80.1	73.2	0.08	1.8	0.91
**Child nutrition (child 6–11 months)**								
Child eats solid or semisolid food	48.0	52.0	0.37	66.8	82.7	<0.01	11.8	<0.01
Child began eating solid food by age 6 months	27.4	30.4	0.56	42.1	55.4	<0.01	10.6	0.12

Carmichael SL, *et al. BMJ Global Health* 2019; 4:e001146. doi: 10.1136/bmjgh-2018-001146

Appropriate frequency of feeding in previous day[e]	20.6	22.5	0.70	32.3	44.0	<0.01	9.4	0.27
**Immunizations (child 6–11 months)**								
Received measles vaccine (for children 9–11 months)	33.4	34.6	0.57	40.2	68.4	<0.01	26.9	<0.01

[a] Survey-weighted percentages and counts are reported, to account for the survey design. Survey logistic regression models were performed that accounted for village as the primary sampling unit and sub-center as the primary stratum within the sampling unit, with proportional sampling weights at the maternal respondent/household level; each model also included maternal age (as a continuous variable) and caste (non-Hindu, Hindu SC/ST, Hindu not SC/ST).

[b] Separate regression models were conducted for each outcome, at baseline and post-implementation; p-values reflect comparisons of the intervention and control groups, at each time point.

[c] In order to estimate the effect of the TBGI intervention on a particular indicator, we conducted a separate regression model for each outcome that included all maternal respondents. These models each contained a term representing time (baseline or post-implementation), a term representing treatment (intervention or control), and an interaction of these two terms, which is represented by the difference in difference estimator (DID) and its p-value. The DID reflects treatment effects (positive values reflect the amount of improvement attributable to the intervention).

[d] Based on reports of liquids and solids fed to children younger than 6 months old in the previous 24 hours, following the recommended definition of the World Health Organization

[e] Defined as 2 or more times for children 6-8 months old and 3 or more times for children 9-11 months old.

Carmichael SL, *et al. BMJ Global Health* 2019; 4:e001146. doi: 10.1136/bmjgh-2018-001146
